# Moderators and Other Predictors of Methylphenidate Response in Children and Adolescents with ADHD

**DOI:** 10.3390/ijerph19031640

**Published:** 2022-01-31

**Authors:** Barbara D’Aiello, Silvia Di Vara, Pietro De Rossi, Italo Pretelli, Stefano Vicari, Deny Menghini

**Affiliations:** 1 Child and Adolescent Neuropsychiatry Unit, Department of Neuroscience, Bambino Gesù Children’s Hospital, IRCCS, 00146 Rome, Italy; barbara.daiello@opbg.net (B.D.); silvia.divara@opbg.net (S.D.V.); pietro.derossi@opbg.net (P.D.R.); italo.pretelli@opbg.net (I.P.); stefano.vicari@opbg.net (S.V.); 2 Department of Human Science, LUMSA University, 00193 Rome, Italy; 3 Department of Life Science and Public Health, Università Cattolica del Sacro Cuore, 00168 Rome, Italy; 4 Centro di Riabilitazione, Casa San Giuseppe, Opera Don Guanella, 00165 Rome, Italy

**Keywords:** methylphenidate, ADHD, behavioral and emotional symptoms, executive functions, conduct symptoms

## Abstract

Methylphenidate (MPH) is the treatment of first choice for developmental ADHD. To date, no reliable method to predict how patients will respond to MPH exists and conflicting results are reported on clinical characteristics of responders. The present study aims to give a more precise characterization of the patients who will respond best to MPH to help clinicians in defining the treatment plan. Age, neuropsychological functioning (i.e., attention and working memory), and behavioral/emotional symptoms of 48 drug-naïve children and adolescents with ADHD (42 boys and 6 girls, age-range 6–16 years, mean age 10.5 ± 2.5 years, mean IQ 101.3 ± 11.2) were studied to assess how these different characteristics affected a single-dose MPH response. Four hierarchical linear regression models were used to explore whether age, neuropsychological measures at baseline, and behavioral/emotional symptoms could predict attention and working memory measures after a single-dose MPH administration. We found that improvement in attention and working memory was predicted by age, neuropsychological measures at baseline, and severity of ADHD symptoms. No behavioral and emotional symptoms predicted single-dose MPH response with the exception of conduct symptoms.

## 1. Introduction

Executive functions (EF) are cognitive processes that allow problem-solving behavior geared toward the attainment of a future goal [1]. Several studies [2,3,4] have documented that a set of EF, including response inhibition and working memory, are deficient in attention-deficit hyperactivity disorder (ADHD). Specifically, working memory deficits are found in 30% to 37% of children with ADHD [4,5] and inhibitory control deficits in 21% to 46% [4,6,7,8].

Studies have demonstrates that patients with ADHD improve significantly in EF when on methylphenidate (MPH), the first-choice treatment for ADHD in the developmental age [9,10]. In particular, MPH ameliorated inhibitory control [4,11,12,13,14], visual-spatial working memory [15], sustained and selective attention [16], and reaction times (RT) [14].

A recent meta-analysis [17] suggests that after a flexible titration, i.e., considering the presence of ADHD symptoms, and tolerated, i.e., considering the presence of dose-limiting adverse effects, higher doses of stimulants were associated with both better efficacy and acceptability.

There is evidence that when MPH dosage was optimized the majority of patients with ADHD achieved a remission of symptoms and demonstrated functional improvement attaining to the level of non-ADHD peers [18]. However, there is also evidence that the severity of ADHD and comorbid symptoms, such as conduct problems, oppositional and defiant behavior, depression, and substance use may interfere with MPH effect [19,20,21,22]. A recent study [23] demonstrated that emotional and behavioral-associated symptoms influenced pharmacological response in ADHD. Specifically, children with ADHD and comorbid emotional dysregulation responded worse to a 4-week MPH administration than children with ADHD without associated symptoms.

There has been a growing interest in identifying predictive factors of response to pharmacological treatment in ADHD. Besides comorbid psychopathology that could worsen the MPH response, pre-treatment EF measures have been recently found as predictors of the clinical response to MPH [24]. Age has also been considered as a possible mediator of MPH effect [25]. Neuroimaging studies [12,26] observed that the effect of MPH was stronger in younger children with ADHD than in older ones. Nevertheless, the majority of studies did not take age into account as a possible moderating factor of MPH response, and the few studies that considered age as a possible confounding factor lead to mixed results [19,24]. 

The aim of the present study is to better understand what factors condition the response to MPH in children and adolescents with ADHD.

In this context, we examine how different aspects of drug-naïve children and adolescents with ADHD, such as age, EF (i.e., attention and working memory) at baseline, and behavioral/emotional symptoms impact on EF changes after a single dose of MPH administration.

We hypothesize that the greater the severity of ADHD, behavioral and emotional symptoms, age of the participants, attention and working memory deficits, the worse the response will be to the MPH administration.

## 2. Materials and Methods

### 2.1. Participants

Three hundred and two children and adolescents received a first diagnosis of ADHD at the Child and Adolescent Neuropsychiatric Unit of the Bambino Gesù Children’s Hospital in Rome in 2020. Among these patients, 110 were prescribed MPH for the first time and underwent a single MPH dose challenge. 

We excluded from the current study 62 patients for the following reasons: (1) they were under psychopharmacological treatment different from MPH at the time of recruitment; (2) they suffered from autistic spectrum disorder; (3) they suffered from genetic syndromes; (4) they suffered from neurological disorders; and (5) they had an IQ below 80.

According to the exclusion criteria, 48 participants with the combined hyperactive/impulsive and inattentive presentation of ADHD were recruited (Table 1). Neurocognitive performance may differ between ADHD presentations [27]. Therefore, including only participants with ADHD combined presentation and not participants with the inattentive or hyperactive/impulsive presentation, might increase homogeneity in neurocognitive functioning [25].

After performing the neuropsychiatric and psychopathological assessment, each participant completed neuropsychological tasks at baseline (t0) and after MPH administration (t1). All participants and parents were informed about assessment instruments and treatment options. Written informed consent was obtained from parents. The study was conformed to Declaration of Helsinki.

### 2.2. Psychopathological Assessment

Psychiatric diagnoses were based on developmental history, extensive clinical examination and the Schedule for Affective Disorders and Schizophrenia for School-Aged Children Present and Lifetime Version DSM-5 [28], a semi-structured interview that assesses the presence of psychopathological disorders according to DSM-5 classification. 

Behavioral and emotional symptoms were assessed by the child behavior checklist 6–18 (CBCL) and the Achenbach system of empirically based assessment (ASEBA) questionnaire. The CBCL parent questionnaire [29] is a well-known tool to detecting psychopathological symptoms in children and adolescents. The hierarchical structure of the CBCL encompasses several scales. We analyzed the following six DSM-oriented Scales (CBCL affective problems, anxiety problems, somatic problems, ADHD problems, oppositional defiant problems, and conduct problems) because there were no overlapping items across scales. Raw scores were converted in T-scores. According to the cut-off thresholds of Achenbach and Rescorla [29], T-scores > 69 were classified as clinically relevant, T-scores between 65 and 69 were classified as borderline, and T-scores < 65 indicated non-clinical symptoms.

The severity of ADHD symptoms was assessed by Conners’ parent rating scales long version revised (CPRS) [30], completed by parents. We analyzed two DSM-IV symptom scales: inattentive (CPRS L) and hyperactive-impulsive (CPRS M). Raw scores were converted in T-scores. According to the cut-off thresholds, T-scores >70 were classified as very elevated and T-scores from 60 to 70 were classified as high average or elevated.

### 2.3. Executive Functions Assessment 

The continuous performance test II (CPT) [31] is composed of 360 letters, presented one at a time for approximately 250 ms each, which are presented in the standard format of 18 sub-blocks of 20 trials each. The blocks differ in the interstimulus intervals (ISI) between letter presentations, which last 1, 2, or 4 s. ISI are randomized between blocks so that all three ISI conditions occur every three blocks. The transition from one block to the next is unannounced and occurs without delay. The participants were instructed to press the spacebar when any letter except the letter “X” appeared on the screen. The percentage of trials when letters other than “X” appear is 90% across all ISI blocks. RT is measured from the point at which any letter other than “X” appears on the screen until the spacebar was pressed (Go trial). No-Go trials occur when an “X” is presented. The task took 14 min to complete. All participants had a 3-min practice session prior to starting the CPT, to reduce the effect of familiarity stemming from repeated tests. Accuracy, reaction times in milliseconds, and the reaction time variability (RTV) in milliseconds were recorded at t0 and t1.

The N-back [32] is one of the most widely used culture free tools applied to evaluate working memory. The visual-spatial condition consists of presenting a series of visual stimuli (blue boxes) in a certain location on the screen. After a training phase, participants were required to indicate whether the location of each box presented was the same as the location of the box presented immediately prior (level: 1-back). When the accuracy was equal to or greater than 80%, the difficulty of the N-back increased (for example, passing from 1-back to 2-back). The analyses were based on scores in the last N-back span achieved (i.e., percentage accuracy value ≥80%) and the percentage of accuracy in the next unachieved N-back (i.e., percentage accuracy value <80%). For example, when the participant reached the 2-back and its accuracy exceeded only 30%, the score was 2.3.

### 2.4. Medication 

MPH is the first-line medication for children and adolescents with ADHD in line with the National Institute for Health and Care Excellence (NICE) and Agenzia Italiana del Farmaco (AIFA) guidelines. Before the single-dose MPH challenge, all the patients who were eligible to receive MPH treatment underwent an electrocardiogram (ECG) with the calculation of the corrected QT interval, and blood tests to exclude any other medical condition associated with ADHD or potentially mimicking ADHD symptoms (e.g., thyroiditis). All participants underwent CPT and N-back one day before drug administration (t0) and one hour after the administration of 0.3 mg/kg of the short-acting MPH preparation Ritalin© (t1). 

### 2.5. Statistical Analyses

Paired sample *t*-tests were used to compare EF measures at t0 and at t1. To correct for multiple comparisons (4 measures: CPT Accuracy, CPT RT, CPT RTV, and N-back scores), Holm–Bonferroni-corrected alpha values were applied [33]. 

To determine whether MPH response was predicted by age, EF measures at t0 and behavioral and emotional measures, four different hierarchical regression analyses with 3 steps were computed. Precisely, the dependent variables were EF measures (N-back scores, CPT Accuracy, CPT RT, or CPT RTV) at t1 and the predictors were age at step 1, EF measures at t0, the two DSM-IV symptoms scales of CPRS (inattentive and hyperactive-impulsive) at step 2, and the six DSM-oriented Scales of CBCL (affective problems, anxiety problems, somatic problems, ADHD problems, oppositional defiant problems, and conduct problems) at step 3. Moderation effects were examined using multicollinearity tests for interaction.

The statistical software SPSS Version 22 (IBM Corporation, Armonk, NY, USA, 2017) was used for analyses.

## 3. Results

Concerning working memory, results on N-back demonstrated that the scores obtained by children and adolescents with ADHD at t0 were lower than scores obtained at t1 (t_47_ = −2.35, *p* = 0.023, Cohen’s d = 0.44) as reported in Table 2.

As for CPT, we found that participants improved CPT accuracy (t_47_ = −4.71, *p* < 0.001, Cohen’s d = 0.51) and reduced CPT RTV (t_47_ = 0.45, *p* < 0.001, Cohen’s d = 0.56) at t1 compared to t0. CPT RT did not differ between t0 and t1 (t_47_ = 0.96, *p* = 0.33, Cohen’s d = 0.13). 

After the Holm–Bonferroni correction, N-back scores (*p* = 0.04), CPT accuracy (*p* = 0.004), and CPT RTV (*p* = 0.004) were still significant.

In the first model of the forward hierarchical regression to predict N-back scores at t1, age was entered at step 1, N-back scores at t0, the two DSM-IV symptoms scales of CPRS (inattentive and hyperactive-impulsive) were entered at step 2, and the six DSM-oriented scales of CBCL (affective problems, anxiety problems, somatic problems, ADHD problems, oppositional defiant problems, and conduct problems) were entered at step 3 as predictors. Overall, the regression model accounted for 51.9% of the variance. As reported in Table 3, age accounted for 29.2% of the unique variance (with older children improving more), while the N-back scores at t0 accounted for 7.4% (with higher scores at t1 in participants who demonstrated higher scores at t0). 

Moreover, CPRS M accounted for 5.5% of the unique variance (with lower scores for higher N-back scores at t1) and CBCL ADHD problems accounted for 9.8% of the unique variance (with lower scores for higher N-back scores at t1). No interaction effect was found between any of the predictive variables.

In the second model of forward hierarchical regression to predict CPT accuracy at t1, age was entered at step 1, CPT accuracy at t0, the two DSM-IV symptoms scales of CPRS (inattentive and hyperactive-impulsive) were entered at step 2, and six CBCL DSM-oriented scales (affective problems, anxiety problems, somatic problems, ADHD problems, oppositional defiant problems, and conduct problems) were entered at step 3 as predictors. Overall, the regression model accounted for 51.8% of the variance. As reported in Table 4, the age accounted for 22.4% of variance (with older children improving more), and the CPT accuracy at t0 accounted for 29.5% (with higher scores at t1 in participants who demonstrated higher scores at t0). No significant effect of CBCL DSM-oriented scales or of DSM-IV symptoms scales of CPRS on CPT accuracy at t1 was found.

In the third model of the forward hierarchical regression to predict CPT RT at t1, age was entered at step 1, CPT RT at t0, the two DSM-IV symptoms scales of CPRS (inattentive and hyperactive-impulsive) were entered at step 2, and the six DSM-oriented scales of CBCL (affective problems, anxiety problems, somatic problems, ADHD problems, oppositional defiant problems, and conduct problems) were entered at step 3 as predictors. Overall, the regression model accounted for 41% of the variance. As reported in Table 5, age accounted for 10% of variance (with younger children improving less), the CPT RT at t0 accounted for 20.7% (with higher scores at t1 in participants who showed higher scores at t0) and the CBCL conduct problems scale accounted for 10.3% (with higher scores for higher CPT RT at t1). No interaction effect was found between any of the predictive variables.

Similarly, in the forward hierarchical regression to predict CPT RTV at t1, age was entered at step 1, CPT RTV at t0, the two DSM-IV symptoms scales of CPRS (inattentive and hyperactive-impulsive) were entered at step 2, and the six CBCL DSM-oriented scales CBCL (affective problems, anxiety problems, somatic problems, ADHD problems, oppositional defiant problems, and conduct problems) were entered at step 3 as predictors. Overall, the regression model accounted for 48.5% of the variance. As reported in Table 6, the age accounted for 26.4% of variance (with younger children improving less), and CPT RTV at t0 accounted for 22% (with higher scores for higher CPT RTV at t1). The results did not show any significant effect of the CBCL DSM-oriented scales or DSM-IV symptoms scales of CPRS on CPT RTV at t1.

## 4. Discussion

This study aimed at a better characterization of different factors potentially associated with MPH response. Specifically, we explored whether age, EF measures at baseline, and behavioral/emotional symptoms could affect EF in a group of drug-naïve children and adolescent with ADHD after a single dose of MPH administration. We found that attention and working memory improved after a single MPH administration and that age, EF measures at baseline, the severity of ADHD symptoms, and conduct problems modulated MPH effect on EF performances. 

MPH is the first-choice treatment for patients with ADHD. Although its exact mechanism is unclear, it seems to increase and stabilize catecholaminergic neurotransmission in prefrontal cortices [10]. The activity of prefrontal cortices affected by MPH participated in basically all of the EF [4,9], as the most impaired functions in ADHD [34].

Neuroimaging studies demonstrated that EF deficits were highly related to reduced activity in fronto-striatal and fronto-parietal networks of patients with ADHD [35]. In particular, deficits in inhibitory control were linked to abnormalities in the right-hemispheric fronto-basal ganglia networks, including the right inferior frontal gyrus and striatal regions [36,37], and deficits in working memory were associated with the decreased efficiency of the dorsal lateral prefrontal cortex. Thus, one may speculate that MPH, acting on prefrontal networks, could induce a positive effect on EF [38], ameliorating cognitive and behavioral deficits of children with ADHD [39]. These ameliorative effects on EF should be considered as indications of improvement due to the psychostimulant medication in children with ADHD [40,41].

Our results on attention and working memory improvements after a single MPH administration supported previous findings observing that MPH significantly ameliorating attention by reducing RT [19,42], and promoted the updating of information in visual-spatial working memory [15].

Moreover, we found that higher scores in N-back and CPT tasks at baseline favored performance in N-back and CPT after a single dose of MPH administration. Previous research in children with ADHD studied whether the performances on EF tasks before MPH administration were correlated with responses to MPH [19,43,44] with contrasting outcomes. While some studies observed that lower scores on neuropsychological tasks at baseline predicted higher responses to MPH [19,43,44], others observed that MPH did not modify the performance on tasks with executive components [45] or still others demonstrated that children with ADHD who had lower scores in tasks as CPT were less likely to respond to MPH [46,47]. We cannot easily compare our results with the previous ones because we studied the effects on EF of a single dose of MPH while the other studies evaluated long-term drug treatment effects (lasting at least 3 months).

Regarding age, we found that improvement in attention and working memory after a single dose of MPH administration was predicted by age. Specifically, older children with ADHD were those who got the most benefit from a single dose of MPH administration in EF (i.e., in N-back scores, CPT Accuracy, RT, and RTV). It could be that as patients with ADHD develop, they learn to solve complex problems with increasing accuracy, reducing the variability in the performance and shortening times to information processing. Specifically, the performance of patients with ADHD in shifting attention (correct responses and errors) improved with age and children below 10 years old were less respondent to MPH [46].

Additionally, our results demonstrated that the MPH effect on working memory (N-back) depended on the severity of ADHD symptoms. In particular, we found that the children and adolescents who demonstrated less severe symptoms of ADHD (in the CPRS hyperactive-impulsive scale and CBCL ADHD scale) were the ones who improved the most in N-back scores. Our findings were in line with studies that found that patients with more severe ADHD symptoms also responded less to stimulants [23,48]. 

Among emotional and behavioral symptoms that may influence the MPH response, our results demonstrated that patients with more conduct problem symptoms (CBCL conduct problems) responded more to stimulants (CPT RT). Our findings are in line with previous studies demonstrating that children with ADHD and conduct problems respond better to medication [19,24,49,50]. This may likely be due to the fact that MPH is also effective in behavioral disorder with aggressive behaviors [51]. In the current study, no other emotional and behavioral symptoms from CBCL-mediated the MPH response. 

However, it is difficult to compare our results with other studies, because the same CBCL scales to predict MPH response were not selected. Specifically, Ludwig and colleagues [52] selected the sluggish cognitive tempo scale to measure the response to MPH in children with ADHD, while Masi and colleagues [23] selected the dysregulation profile from the CBCL syndrome scales (i.e., anxious/depressed, attention problems, and aggressive behavior). 

Further studies are needed in order to establish whether and how clinical characteristics impact pharmacological treatment for ADHD.

## 5. Conclusions

In the present study of a group of medication-naïve children and adolescents with ADHD, we investigated whether age, EF measures, and clinical characteristics before MPH administration influenced response to medication.

We found that improvement in attention and working memory performance after a single administration of MPH were predicted by age, EF measures, and severity of ADHD symptoms. Moreover, we found that children and adolescents with ADHD and conduct symptoms improved EF more than patients with ADHD and other symptoms after a single dose of MPH.

Early attention to these factors may help clinicians identify young patients with ADHD who are likely to gain greater benefit from MPH treatment, thereby optimizing the risk/benefit ratio in the pharmacologic treatment of ADHD.

## Figures and Tables

**Table 1:** Demographic information of participants with ADHD.

Demographic Characteristics	*N*	Mean (SD)	% of Total Sample
Gender			
Males	42		
Females	6		
Age		10.5 (2.5)	
IQ		101.3 (11.2)	
Comorbid diagnosis			
Oppositional defiant disorder			43.8
Specific learning disorder			25
Anxiety disorder			8.3
No comorbid diagnosis			22.9

**Table 2:** Comparisons between t0-t1 on neuropsychological measures.

Measures	t0 Mean (SD)	t1 Mean (SD)
N-back	1.6 (0.4)	1.8 (0.5)
CPT accuracy	92.6 (6.2)	95.5 (5.1)
CPT RT	448.1 (96.1)	435.9 (80.5)
CPT RTV	285.8 (154.3)	206.41 (127.2)

**Table 3:** Hierarchical linear regression model predicting N-back at t1 after MPH administration.

Steps	Predictors	R^2^	F	*p*	*B*
Step 1	Age	0.292	19.0	0.0001	0.26
Steps	N-back at t0	0.074	5.2	0.027	0.31
	CPRS M	0.055	4.2	0.046	−0.07
Step 3	CBCL ADHD problems	0.098	8.7	0.005	−0.36

**Table 4:** Hierarchical linear regression model predicting CPT accuracy at t1 after MPH administration.

Steps	Predictors	R^2^	F	*p*	*B*
Step 1	Age	0.224	13.2	0.001	0.04
Step 2	CPT accuracy at t0	0.295	27.5	0.0001	0.69

**Table 5:** Hierarchical linear regression model predicting CPT RT at t1 after MPH administration.

Steps	Predictors	R^2^	F	*p*	*B*
Step 1	Age	0.100	5.1	0.028	−0.13
Step 2	CPT RT at t0	0.207	13.4	0.001	0.43
Step 3	CBCL conduct problems	0.103	7.6	0.008	−0.32

**Table 6:** Hierarchical linear regression model predicting CPT RTV at t1 after MPH administration.

Steps	Predictors	R^2^	F	*p*	*B*
Step 1	Age	0.264	16.5	0.0001	−0.27
Step 2	CPT RTV at t0	0.220	19.2	0.0001	0.52

## Data Availability

The data presented in this study are available on request from the corresponding author. The data are not publicly available due to privacy and ethical restrictions.
